# Supplementary figures and images for: Mitonuclear Sex Determination? Empirical Evidence from Bivalves
Source: Mol Biol Evol. 2023 Nov 3;40(11):msad240. doi: 10.1093/molbev/msad240 (PMC10653589; doi:10.1093/molbev/msad240)

ranked\_0

unit: Angstrom

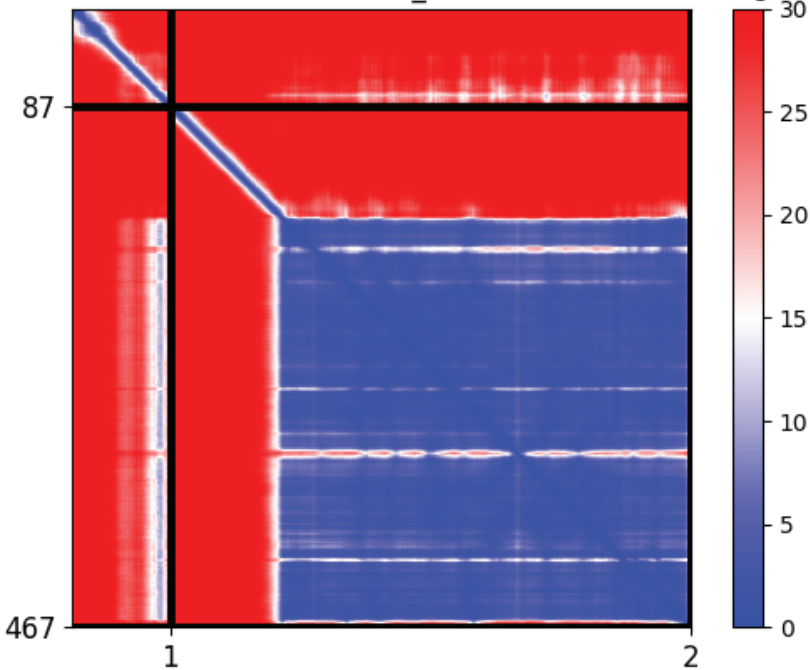

Supplement: msad240_Supplementary_Data [file msad240_supplementary_data.zip › Supplemental Figures/FigureS1.pdf]

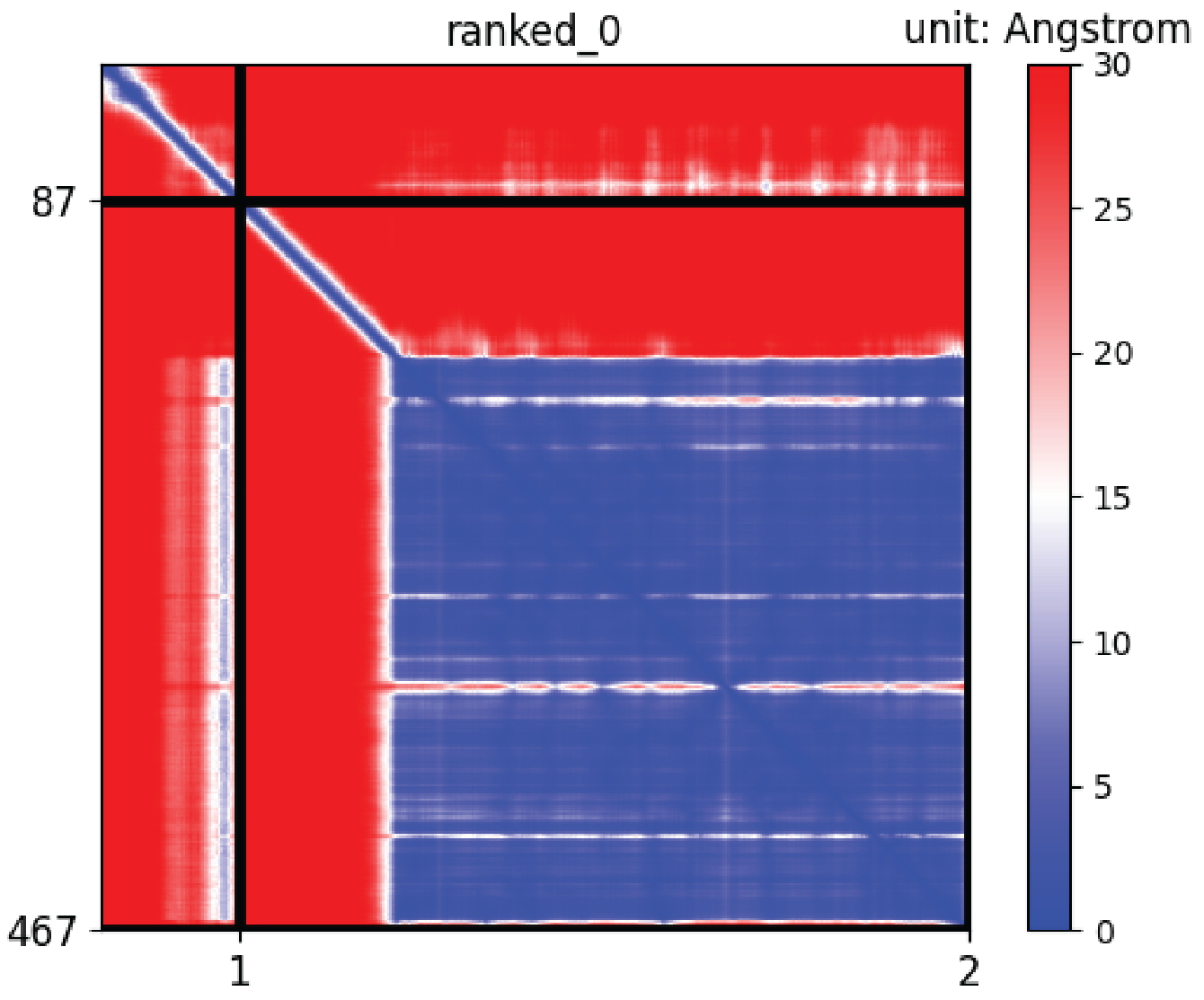

Supplement: msad240_Supplementary_Data [file msad240_supplementary_data.zip › Supplemental Figures/FigureS1.png]
